# The Resistance to Freeze-Drying and to Storage Was Determined as the Cellular Ability to Recover Its Survival Rate and Acidification Activity

**DOI:** 10.1155/2010/625239

**Published:** 2010-06-16

**Authors:** Ibourahema Coulibaly, Robin Dubois-Dauphin, Jacqueline Destain, Marie-Laure Fauconnier, Georges Lognay, Philippe Thonart

**Affiliations:** ^1^Wallon Center for Industrial Biology, Bio-Industry Unit, Gembloux Agricultural University, Passage des déportés 2, 5030 Gembloux, Belgium; ^2^Analytical Chemistry Unit, Gembloux Agricultural University, Passages des déportés 2, 5030 Gembloux, Belgium; ^3^Plant Biology Unit, Gembloux Agricultural University, Passages des déportés 2, 5030 Gembloux, Belgium; ^4^Wallon Center for Industrial Biology, Microbial Technology Unit, University of Liège, Sart-Tilman B40, 4000 Liège, Belgium

## Abstract

The protective effects of the fatty acid composition and membrane action of the acidification activity of two strains of *Lactobacillus* kept at 20°C were studied. The addition of sorbitol, monosodium glutamate and glycerol during storage is causing the decline of acidification and increased concentrations of unsaturated fatty acids observed in both strains. The addition of sorbitol and monosodium glutamate does not alter the fatty acid composition, whatever the strain, but increases the resistance to freeze-drying of *L. plantarum* CWBI-B1419 and improves survival during storage. The addition of these preservatives and decreased activity of acidification improves the ratio unsaturated. These results indicate that the survival during storage and freeze-drying resistance are closely related to the composition of membrane fatty acids. This behaviour can be interpreted as an adaptation of *L. plantarum* B1419-CWBI supplemented by cryoprotectant additives such as sorbitol or monosodium glutamate sorbitol and monosodium glutamate as an additive. *L. plantarum* CWBI-B1419 presents a greater adaptation to culture conditions than *L. paracasei* ssp. *paracasei *LMG9192^T^.

## 1. Introduction

The acidification activity of lactic acid bacteria at the different steps of their production (fermentation, cooling, concentration, cryoprotection, freezing, or freeze-drying) and during storage differs depending on the strain considered and on the operating conditions [1, 2]. The freezing and freeze-drying step is especially critical as it negatively affects both viability and physiological state of the bacteria [3, 4]. The formation of ice crystals induces mechanical damage that leads to cellular death during freezing [5]. In addition, the crystallization of the water leads to a cryoconcentration of the solutes, which induces some osmotic damage [6]. Some agents such as those used in lyoprotection are of undoubted importance for the survival of cells because they can act on biological functions in preserving the integrity of the lipid bilayer by the phenomenon of water replacement, vitrification (glass formation), and depression of membrane transition temperatures (Tm), as reviewed in detail by Castro et al. [7, 8] and Champagne et al. [9]. The same observations with sucrose for survival of *Lb. coryniformis* Si3 were noticed by Schoug et al. [10]. Adding cryoprotective agents such as sorbitol, monosodium glutamate, and glycerol before freeze-drying process attenuated the damaging effects of freezing, thus improving the bacterial resistance to drying [11, 12]. This protective effect was ascribed to interactions between sorbitol and the membrane phospholipids during the first step of freeze-drying, freezing [13]. Because the cell membrane is the first target to modification of the cell environment, its ability to adapt largely determines the survivability of the cell [14, 15]. By considering the important role of fatty acid organization in membrane permeability, the membrane viscosity [16] and the membrane thickness [17] were ascribed to the unsaturation index of membrane fatty acids: the cell membrane adapts by increasing the proportion of unsaturated fatty acids, [18–20]. Unsaturated fatty acids promote exchanges between extracellular and intracellular media by rigidifying the membrane and enhancing the membrane permeability. The increased membrane permeability is related to the presence of the double bounds that tend to form less stable Van-der-waals interactions with adjacent lipids [17]. As a consequence, altering the fatty acid composition of the membrane may improve membrane permeability at low temperature and then may allow the cell to adapt itself to freezing and freeze-drying [21]. They considered either the concentration in some unsaturated fatty acids, or the ratio between unsaturated and saturated fatty acids (U/S). The U/S ratio depends on the medium and environmental conditions in which the cells are cultivated and stored. Concerning lactic acid bacteria, the addition of ethanol or polyol such as sorbitol in the culture medium enhances the concentration in dihydrosterculic acid and the U/S ratio [22, 23]. The biosynthesis of unsaturated C_18:1_ fatty acids by some lactic acid bacteria is stimulated by the addition of ethanol and leads to an increase of the U/S ratio [24]. Finally, the fatty acid composition evolves during storage. Castro et al. [7] observed two phases: a first increase of the U/S ratio, which is explained by lipolysis reactions, followed by a decrease. Linders et al. [25] showed that U/S ratio is stable within 90 d of storage and then decreases. This decrease is linked to the oxidation of unsaturated fatty acids that are very sensitive to oxygen [7] and is accentuated by an increase in the residual relative humidity that activates the oxidation processes [8]. It is clear that acting on the membrane fatty acid composition can modulate the U/S ratio. This was achieved by using appropriate operating conditions and led to a better recovery of cellular viability after freeze-drying and subsequent storage [26]. Nevertheless, as viability measurements are insufficient to express both viability and physiological states of lactic acid bacteria, these have to be proved by considering the acidification activity of lactic acid bacteria. This work aimed to characterize the survival rate, resistance, and subsequent storage of a freeze-dried *Lactobacillus plantarum* strain is in relation to its fatty acid composition. The resistance to freeze-drying and to storage was determined as the cellular ability to recover its survival rate and acidification activity.

## 2. Materials and Methods

### 2.1. Microorganisms and Growth Conditions

#### 2.1.1. Growth Conditions

*Lactobacillus plantarum *CWBI-B1419 was obtained from the collection of the Wallon Center for Industrial's Biology, Belgium and was originally isolated from a poultry farm. *Lactobacillus paracasei *ssp. *paracasei *LMG9192^T^ was obtained from the culture collection of the Laboratory of Microbiology of Ghent's University (Belgium). The original reference cultures were maintained in cryogenic storage on glass beads at −80°C. Working cultures were maintained as slopes on de Man-Rogosa-Sharp agar (MRSa). Slopes were stored at 4°C and subcultured every month. *L. plantarum *CWBI-B1419 and* L. paracasei *ssp. *paracsei *LMG9192^T^ were grown in MRS medium (Merck) at 37°C. The inoculum of the tested bacteria was prepared from strains stored on a substrate with glycerol at −80°C using appropriate media. Bacteria were proliferated for 24 hours and further culturing was carried out after increasing the volume of the medium for the next 48 hours anaerobically at 37°C. Bacteria prepared in this way provided the inoculum which was used at the amount of 1% (v/v).

#### 2.1.2. Scaling Up

Strains were grown and performed in 100 L bioreactor at 37°C containing MRS medium for 18 hours and then concentrated 20 times by centrifugation. The pH was controlled at 6.5 by adding a 14% KOH. Cells were freeze-dried in a Low freeze-drier (Leybold, Belgium) with a standard programme by increasing the temperature gradually from −45°C to 25°C at 0.9 mbar pressure (30 hours) followed by 15 hours at 0.15 mbar. All fermentations were done in duplicate and average values reported. 

#### 2.1.3. Cells Concentrations and Storage

Cells were harvested in the stationary phase of growth by centrifugation (7000 × *g*, 30 minutes, 4°C). Concentrated cells were resuspended in the same weight of supernatant, at 4°C. Washed cells were then resuspended in sterile Ringer's solution with (3% w/w) of glycerol containing selected concentrations of compounds to be tested: 12 g/L sorbitol (s), and 12 g/L monosodium glutamate (msg), (Sigma, St. Louis, MO, USA). Protectant solutions were sterilized at 121°C for 10 minutes before mixing with a volume of washed cells. Cellular suspensions were maintained for 1 hour at room temperature before freeze-drying, in order to create an isotonic environment between cells and the compound added. Pastes were freeze-dried in trays, using an industrial freeze-dryer (HETO, Heto-Holten A/S, Allerd, Denmark). The initial pressure was 0.2 mbar, the plate temperature was −45°C and the end temperature of the product was 25°C. Freeze-drying time varied according to the amount of material in the drying room and was approximately 18–30 hours. Dried powders were stored in closed container at −20°C for one night without light. Freeze-dried powders are divided in two parts: the first one of about 2 ± 0.5 g was sealed in 15 mL Falcon tubes and stored at room temperature (25°C) without light for 8 months. The second part was vacuum-sealed in aluminium foil and stored at room temperature in darkness.

### 2.2. Rehydration/Enumeration

Viable counts were made before freeze-drying, after drying, and during storage at regular intervals. After freeze-drying, samples were immediately brought to their original volume 9 mL with each peptoned water at 25°C. Then, samples were homogenized for 1 minute by Vortex mixer (SA-5, Stuart Scientific, UK) and incubated at room temperature for 15 minutes. Serial dilutions were spread-plated on the surface of petri plates containing MRS agar. These plates were incubated at 37°C for 48 hours, and the viability was then determined by the drop count technique. Survival levels were expressed as the ratio of colony-forming units per millilitre (cfu/ml) on MRS agar before (N_0_) and after (N_f_) freeze-drying. Viability (%) = (N_F_/N_0_) × 100. 

### 2.3. Acidification Activity and Water Content

Acidification was carried out at 30°C in 120 mL MRS [27] broth inoculated with 1% of 10^7^ Cfu·ml^−1^ of the freeze-dried sample. The total titratable acidity (% lactic acid/g DW) was determinate after 18 hours, according to the AOAC method (1997). The water content (g H_2_O/100 g dry weight) of the freeze-dried samples was determined after drying at 105°C until constant weight. Water activities of the dried samples after freeze-drying and the saturated salt solutions were measured and confirmed with a Novasina (Novasina, Pfäffikon, *Switzerland*) water activity meter (Aqua Lab, CX-2, Decagon Devices, INC, Washington, U.S.A.) by the dew point method with a standard deviation SD ± 0.003, respectively. Residual water content was <3% in all samples.

### 2.4. Fatty Acids Analyses (FAME)

Total lipids extraction was performed according to the adapted method of [28]. Fatty acid methyl esters were prepared by incubating the lipid extracts at 70°C for 90 minutes in 0. 5 mL of methanol-BF3, containing 15% (v/v) of KOH and 0. 2 mL of hexane. The fatty acids were extracted to the upper phase of regent mixture containing 1 mL of hexane, 0.5 mL of saturated NaCl; H_2_SO_4_ 10% 0.2 mL and analysed by gas chromatography on a HP 6890 (Hewlett Packard, Germany) equipped with a flame ionization detector and a SPTM-2560, 100 m* 0.25 mm* 0.2 *μ*m fused silica capillary column. The conditions were as follows: injector temperature, 260°C; detector temperature, 260°C; carrier gas (helium) flow rate, 3 mL/min. The oven temperature was programmed from 140°C during 5 minutes to 240°C at 4°C/min. For peak identification, standard solution (Sigma) was used. The results were relative percentages of fatty acids, determined from peak areas of methyl esters. They were means of three independent experiments. 

#### 2.4.1. Statistical Analysis

A four-factor analysis of variance with two-factor interactions (*Stastica 9*) was performed to determine the effects of sorbitol, the addition of monosodium glutamate and glycerol, and the length of storage. The Neuman-Keuls multiple comparison procedure was used to discriminate among the means for significant differences at the 5% confidence level. All results presented in this paper are the average of three independent replicate assays. The ratio between the standard deviations and the means values was between 2% and 5%.

## 3. Results and Discussion

### 3.1. Fatty Acid Composition of Freeze-Dried L. plantarum CWBI-B1419 and L. paracasei ssp. paracasei LMG9192^T^

Cellular fatty acids (CFAs) from *L. plantarum *CWBI-B1419 and *L. paracasei* ssp. *paracasei *LMG9192^T^ (control) were analyzed before and after freeze-drying. A total of six fatty acids made up of the membrane of those strains cultivated at pH 6.5, in absence or presence of sorbitol and monosodium glutamate in the washed cells solution (Table 1). This presence or absence of those additives did not affect the number of fatty acid detected or identified. As control U/S ratios were 0.57 and 0.58 for *L. plantarum *CWBI-B1419 and *L. paracasei* LMG9192, respectively; saturated and unsaturated fatty acids were well balanced. When sorbitol and monosodium glutamate were added in the cellular suspensions, the U/S ratio was higher than 0.66 for *L. plantarum* CWBI-B1419 and 0.62 for *L. paracasei* LMG9192, respectively, thus indicating a shift from saturated to unsaturated fatty acids in the membrane composition. This was due to a decrease in C_16:0_, and C_18:0_ and an increase in C_16:1_ and C_18:1_ fatty acids. As confidence intervals overlapped, C_18:2_ and C_18:3_ did not show any significant difference, whether or not additives were present. Except for the C_16:1_, most of these fatty acids have been identified in other species of lactic acid bacteria: *L. lactis *subsp. *lactis *and *Lactobacillus *sp. [23], *Lb. bulgaricus* [8, 29], *Lb. plantarum *[30, 31], *Lb. buchneri *and *Lb. fermentum *[32], *Lactobacillus *sp. [33] and *Leuconostoc mesenteroides *[24].

### 3.2. Impacts of Protective Agents and Storage Time on Membrane Fatty Acid Composition of L. plantarum CWBI-B1419 and L. paracasei ssp. paracasei LMG9192^T^

**Figure 1:**
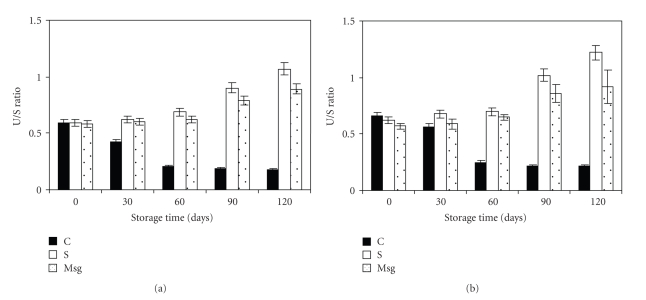
Impact of storage time upon U/S ratio of freeze-dried lactobacilli during 120 days at room temperature in vacuum-sealed aluminium foil. Values are presented as means ± standard deviation (SD, *n* = 4). (a) *L. paracasei *ssp*. paracasei *LMG 9192^T^, (b) *L. plantarum* CWBI-B1419^T^ and (c) control (■), sorbitol (□) and monosodium glutamate (⊡).

The effect of addition of sorbitol, monosodium glutamate, and glycerol as protectives agents and storage time on membrane fatty acid composition was studied. The ratio between unsaturated and saturated fatty acids was more affected by the addition of those additives and the storage time, while the addition of glycerol had no effect. Relative fatty acid concentrations were diversely influenced by glycerol monosodium glutamate and sorbitol. The addition of glycerol as cryoprotective agent did not modify the fatty acid composition of the membrane because this molecule was added after the concentration step, immediately before freeze-drying. At this stage of production, cells were still in a quiet physiological state. Moreover, at this temperature, the membrane permeability is not high [34] and the passive diffusion of the glycerol from extracellular to intracellular medium was clarified. Consequently, glycerol probably acted mainly as an extracellular cryoprotective agent [13]. As expected, the addition of sorbitol and monosodium glutamate increased the concentration of unsaturated fatty acids C_18:1_ and C_16:1_, and decreased the concentration of saturated fatty acids C_16:0_ and C_18:0_ (*P* < .001), without any modification of C_18:1_ and C_18:2_ proportions, (Table 1). The U/S ratio increased from 0.57 to 0.66 with sorbitol and from 0.57 to 0.62 when monosodium glutamate was used. These results agree with those reported by Champagne et al. [9] and Anchordoguy et al. [13] who showed that the U/S ratio is strongly dependent on the presence of polyol in the medium. This indicated that sorbitol, when incorporated in the intracellular medium, increase thus unbalancing of the fatty acid composition. From Table 2, the storage time of the freeze-dried bacteria affected the fatty acid composition (*P* < .05). By increasing the storage time from 0 to 120 days, the C_16:0_ concentration increased, whereas the C_18:1_ and C_18:2_ concentrations decreased (Table 1). Nevertheless, the differences remained slight. In plot of Figure 1, the U/S ratio increased with storage time and according to Table 1, it raised from 0.57 to 0.66, 0.58 to 0.62 with sorbitol, and 0.57 to 0.62 and 0.58 to 0.59 when monosodium was used for *L. plantarum *CWBI-B1419 and* L. paracasei* ssp. *paracasei *LMG9192^T^, respectively. The effect observed on this ratio was more significant as it combined the negative effects that were discerned on the saturated fatty acids and the positive effects noticed on the unsaturated fatty acids. These results agree with those increases during 4 wk of storage of freeze-dried *Lb. bulgaricus*. This was ascribed to lipolysis reactions that altered saturated fatty acid concentrations [13]. The U/S ratio decreased as it was shown for freeze-dried or spray-dried *Lb. bulgaricus *[8, 20]. The oxidation phenomena that were described by these authors were active in the membrane of *L*. *plantarum* CWBI-B1419. In this study, cells were freeze-dried and dehydrated; they were sensitive to oxidation reactions. A significant interaction (*P* < .05) was observed between sorbitol and storage time on the U/S ratio. It could be attributed to a more important effect of the time of storage in the presence of sorbitol and monosodium glutamate in cellular suspensions than when those were absent.

### 3.3. Effects of Sorbitol, Monosodium Glutamate, and Glycerol, on the Survival and Resistance to Freeze-Dried Storage of L. plantarum CWBI-B1419

**Figure 2:**
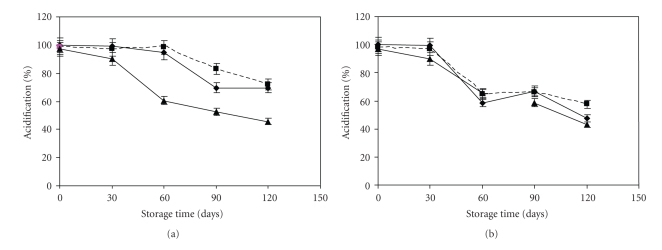
Acidification activity of freeze-dried: (a) *L. plantarum* CWBI-B1419, (b) *L. paracasei *ssp*. casei *LMG 9192^T^ and (c) control (—♦—), sorbitol (—■—) and monosodium glutamate (—▲—). Values are presented as means ± standard deviation (SD, *n* = 4).

**Figure 3:**
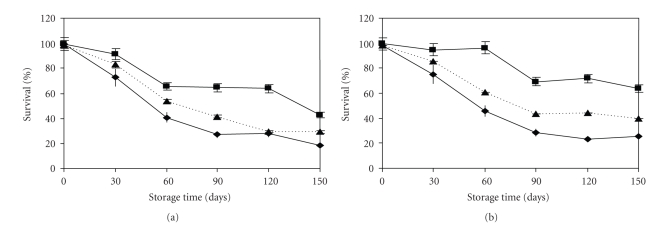
Survival of freeze-dried lactobacilli during 120 d at room temperature in the vacuum seal bags. Values are presented as means ±standard deviation (SD, *n* = 4). (a) *L. paracasei *ssp*. paracasei *LMG 9192^T^, (b) *L. plantarum* CWBI-B1419^T^ and (c) control (—♦—), sorbitol (—■—) and monosodium glutamate (—▲—).

Cryoprotectants (sorbitol and monosodium glutamate) were added to all lactobacilli before being lyophilised powders*. L. plantarum *CWBI-B1419 acidification activity was not significantly different before and after the freeze-drying. During storage of freeze-dried *Lactobacillus*, the acidification activity decreased, as evidenced [11]. In Figure 2 and Table 2, the addition of sorbitol and monosodium glutamate in cellular suspensions showed a significant effect on the rate of loss of acidification activity (*P* < .05). The recovery of the acidification activity was improved by the addition of sorbitol in the resuspended medium (Figure 2). These results are in agreement with those of Simatos et al. [22] who showed that the cellular death of *Lb. bulgaricus *was linearly correlated with the proportion in C_19:0_, and with those of Guerzoni et al. [35], who indicated that *Lactobacillus *sp. and *L. lactis *viability was improved by adding protectants in the culture medium. This could be related to the changes in the fatty acid composition of the cellular membrane that were observed. As expected, the addition of glycerol as a cryoprotective agent considerably improved the resistance to freeze-dried storage of *L. plantarum *(Figure 3). The viability of cells of lactobacilli cultures immediately after freeze-drying was determined. The *L. paracasei *ssp. *paracasei *LMG 9192^T^ strain revealed the lowest percentage of cell recuperation when monosodium glutamate was used as a protective agent.* L. plantarum *CWBI-B1419 was observed to have higher survival than *L. paracasei *ssp.* paracasei *LMG 9192^T^ during storage. It was found good viability of lactobacilli species after drying and subsequent storage when sorbitol and monosodium glutamate were added in cell culture [9]. The rate of loss in acidification activity was three times lower when glycerol was added. This result confirmed the findings of Fonseca et al. [12]. Moreover, the glycerol effect was revealed whatever the other experimental conditions were (presence of sorbitol and monosodium glutamate in cellular suspensions). This indicated that the glycerol displayed an additional effect that can be combined with other beneficial effects. It was found that, survival and cryoprotectants effects of the freeze-dried samples decreased as the storage time increased. However, after the 120 days storage period, a significantly lost of viable population were noted in the freeze-dried samples held at 20°C in vacuum-sealed aluminium foil at 20°C, respectively (*P* < .05) (Figure 3). For example, viable population of freeze-dried lactobacilli held in vacuum-sealed aluminium foil was reduced from an initial population of 7.4 × 10^11^ to 4.77 × 10^9^ or 3.07 × 10^9^ cfu/g with a survival percentage of 64.4% or 41.4% after 120d at 20°C for *L. paracasei *ssp. *paracasei *LMG 9192^T^ with sorbitol, respectively (Table 2). At the same period, survival of *Lactobacillus plantarum *CWBI-B1419 with monosodium glutamate decrease again, after 120 days at 20°C in the same conditions, the viable population was about 8.3 × 10^11^ or 3.57 × 10^9^ cfu/g with a survival of 43.7%, respectively. In addition, viability was significantly higher for freeze-dried strain in vacuum-sealed with sorbitol, a_w_ = 0.11 (*P* < .05). At the end of freeze-drying, the water content of freeze-dried sample was 3.6 ± 0.6 g H_2_O/100 g DW with a_w_ = 0.09 ± 0.01. After the 120 days storage period, the water content for all the samples stored at 20°C was not changed significantly (approximately 0.6-fold) according to the package (*P* < .05). As expected, water content for samples stored under vacuum did not change significantly during storage with sorbitol or monosodium glutamate (*P* < .05), respectively (Table 3).

### 3.4. Relationship between the Resistance to Freeze-Dried L. plantarum CWBI-B1419 Storage and Its Membrane Fatty Acid Composition

The fatty acid composition of *L. plantarum *CWBI-B1419 and the ability of the cells to restore their acidification activity were closely related. The importance of the ratio rate between saturated fatty acids and unsaturated fatty acids results on resistance to freezing and better long-term storage of freeze-dried bacteria [36]. This was observed in all the operating conditions used during the fermentation and freeze-dried storage. Addition of sorbitol or monosodium glutamate to the drying medium indicates a significant change in the survival rate during storage as the confidence level (*P* < .05). Furthermore, the effects were synergistic, except in the case of the existence of interactions [37]. A better resistance to freeze-dried storage was achieved by adding sorbitol and more monosodium glutamate in resuspended medium that led to higher U/S ratios. The positive effect of sorbitol was detected regardless of the fermentation pH and whether or not glycerol was added. Adding sorbitol increased the permeability for water of the membrane, thus favoring transport across the membrane [17]. The ability of monosodium glutamate to protect microorganisms during cryopreservation and freeze-drying has previously been described [38, 39]. The majority of *Lactobacillus* sp. tested and freeze-dried with monosodium glutamate has shown an increased survival during storage. The stabilisation of their protein structure via reactions between the amino group of the protectant and the carboxyl groups of the microorganisms proteins and the ability to retain greater amounts of residual moisture have been pointed out by de Valdéz et al. [40] as explanations that account for protection by monosodium glutamate during freeze-drying and subsequent storage. The cells were then more adapted to suffer the intracellular ice crystallization during freezing and the water mobility during storage [41]. Furthermore, the membrane lipids interact with protein interfaces, either by maintaining the protein structure and activity, or by inhibiting or activating protein functions, such as carrier proteins, which mediate solute transport [9]. For example, the activity of the membrane bound enzyme Na-K-ATPase was shown to be regulated by the lipid portion of the membrane [42]. When sorbitol was added, the effect on the rate of loss in acidification activity could be explained by an influence on some enzymatic activities that may modify the protein composition and content of the cell. This is corroborated by the results of Rallu et al. [43] who reported, in the case of *L. lactis*, an increase in the concentration of cold-shock proteins when the cells suffered a stress. The appearance of unsaturated fatty acids can then be linked to disadvantageous growth conditions [10]. The positive effect of glycerol on the resistance to powders storage is independent of the U/S ratio, which was not affected by this factor [42]. This indicates that two different mechanisms accounted for the different rates of loss in acidification activity: first, a better cellular adaptation, related to the higher water permeability of the membrane, achieved with high U/S ratios; second a cryoprotective effect of glycerol that took place in addition to the previous effects. This interpretation corroborated the previous hypothesis that this molecule probably acted as an extracellular cryoprotective agent. The relationship between the fatty acid composition of *L. plantarum *and its ability to recover acidification activity led to important consequences for performing starter production [25]. From our results, the resistance to freeze-dried storage was improved by increasing the U/S ratio that was obtained by applying unfavorable experimental conditions for growth and by adding sorbitol in the resuspended medium [28]. This concept can be broadened by relating the membrane fatty acid composition to disadvantageous growth conditions, such as low temperature [22, 44, 45] acid stress [43], ethanol stress [45], salt stress osmotic stress [32] or high age of the culture [3, 46]. These results demonstrated that sorbitol has a strong protective effect upon the survival of *L. plantarum *CWBI-B1419 and *L. paracasei* subsp*. paracasei *LMG 9192^T^, during storage, even though no significant differences were observed in terms of viability of cells during freeze-drying in the presence or absence of sorbitol.

## 4. Conclusion

The rate of loss in acidification activity during freeze-dried storage varied according to the conditions in which the cells were cultivated and cryoprotected. The resistance to drying and freeze-dried storage of lactic acid starters was defined by its ability to recover acidification activity after thawing. The resistance to freeze-dried powder during storage was improved by growing at unfavorable conditions and by adding additives (sorbitol and monosodium glutamate) in the resuspended medium and glycerol as protective agent. This improvement was related to an increase of the membrane ratio between unsaturated and saturated fatty acids. The relationship between the U/S ratio and the addition of protectants was obvious. Cryoprotectants such as sorbitol and monosodium glutamate are almost equally effective in protecting lactic acid bacteria dried by freeze-drying. The mechanism of the action of sorbitol and monosodium glutamate appears related to water permeation as sorbitol equally protects cells under hypertonic stress and preserves the osmotic response of the bacteria.

## Figures and Tables

**Table 1:** Composition and modifications of membrane fatty acids (% of peaks areas) induced by growth temperature of *L. paracasei* ssp. LMG 9192^*T*^ and *L. plantarum* CWBI-B1419. The peaks were identified as hexadecanoic (palmitic) acid (C_16:0_), hexadecenoic (palmitoleic) acid (C_16:1_), octadecanoic (stearic) acid (C_18:0_), cis-9-octadecenoic (oleic) acid (C_18:1_), cis-7,12-octadecadienoic (linoleic) acid (C_18:2_) and cis-9,12,15-octadecatrienoic (linolenic) acid (C_18:3_). The hexadecanoic (palmitic) acid (C_16:0_) fatty acids represented almost 50% of the total fatty acids.

Fatty acids	Strains (Percentages of peaks (±7%))					
	LMG 9192^T^			CWBI-B1419		
	Control	Sorbitol	Monosodium glutamate	Control	Sorbitol	Monosodium glutamate
C_16:0_	53.39 ± 0.5	52.90 ± 0.5	53.30 ± 0.4	54.16 ± 0.2	51.60 ± 0.5	52.5 ± 0.3
C_16:1_	7.79 ± 0.1	7.80 ± 0.2	7.20 ± 0.2	9.50 ± 0.1	7.20 ± 0.3	8.74 ± 0.4
C_18:0_	9.80 ± 0.2	9.80 ± 0.4	9.40 ± 0.7	9.58 ± 0.4	8.60 ± 0.2	9.21 ± 0.6
C_18:1_	27.70 ± 0.4	24.9 ± 0.3	26.90 ± 0.1	24.33 ± 0.3	26.40 ± 0.3	26.19 ± 0.7
C_18:2_	0.40 ± 0.02	0.80 ± 0.07	0.70 ± 0.03	0.42 ± 0.05	0.80 ± 0.04	0.76 ± 0.02
C_18:3_	0.90 ± 0.01	5.80 ± 0.2	2.50 ± 0.1	2.01 ± 0.2	5.40 ± 0.6	2.60 ± 0.1

Σ	100	100	100	100	100	100

Σ SFA%	63.20 ± 0.3	62.70 ± 0.7	62.70 ± 0.3	63.74 ± 0.1	60.2 ± 0.5	61.71 ± 0.5
Σ UFA%	36.80 ± 0.5	39.30 ± 0.3	37.30 ± 0.4	36.26 ± 0.2	39.80 ± 0.6	38.29 ± 0.3
U/S	0.58 ± 0.02	0.62 ± 0.02	0.59 ± 0.03	0.57 ± 0.01	0.66 ± 0.03	0.62 ± 0.01

Cells were cultivated by batch fermentation at increased temperatures from 37°C and harvested by centrifugation. After freezing for 24 hours, cells were freeze-dried without cryoprotectant and fatty acids were extracted and analysed by CPG. SFA: Saturated fatty acids; UFA: unsaturated fatty acids;. Data were presented as the average of two independent trials with SD. Note that experiment was not a quantitative method.

**Table 2:** Moisture content and survival of two strains of lactobacilli after freeze-drying with 12 g/L monosodium glutamate and 12 g/L sorbitol. Each value represents the mean of replicates from 2 freeze-drying experiments.

	Vacuum-sealed aluminium foil						
Strains	Moisture content (%)			Initial concentration of dried cells	Survival (%) of dried samples after 120 days storage		
	Control	Sorbitol	Monosodium glutamate	Cfu/g	Control	Sorbitol	Monosodium glutamate
*Lactobacillus* ssp. *paracasei *LMG9192^T^	3.6 ± 0.6^d^	3.4 ± 0.4^b^	3.5 ± 0.3^c^	7.4 × 10^11a^	0.20^d^	0.47^b^	0.31^c^
*Lactobacillus plantarum* CWBI-B1419	3.2 ± 0.5^d^	3.1 ± 0.3^b^	3.4 ± 0.4^c^	8.3 × 10^11a^	0.23^d^	0.57^b^	0.36^c^

Values not sharing the same superscript letter vertical number are significantly different, *P* < .05 (Turkey HSD test, *n* = 4)

^a^Percentage compared those obtained after freeze-drying. Initially, freeze-dried sample with a water content of 3.6 g H_2_O/100 g dry weight contained 7.4 × 10^11^ and 8.3 × 10^11^ *L* *a* *c* *t* *o* *b* *a* *c* *i* *l* *l* *u* *s* ssp**. **paracasei LMG9192^T^ and *L* *a* *c* *t* *o* *b* *a* *c* *i* *l* *l* *u* *s* *p* *l* *a* *n* *t* *a* *r* *u* *m* CWBI-B1419.

^b^Percentage obtained with sorbitol,

^c^monosodium glutamate,

^d^Percentage with control strain. (S): with sorbitol, (msg): monosodium glutamate; C: control (without cryoprotectant).

**Table 3:** Dry cell weight of freeze-dried cells, conserved at 20°C and safe from oxygen and moisture. Water activity of freeze-dried cells, conserved at 20°C and safe from oxygen and moisture.

	Vacuum-sealed aluminium foil					
	Dry cell weight (±1%)			Water activity a_w_ (±0.01)		
Strains	Sorbitol	Monosodium glutamate	Control	Sorbitol	Monosodium glutamate	Control
LMG 9192^T^	98.1 ± 0.89^a^	98.1 ± 0.90	97.98 ± 0.98	0.10 ± 0.01	0.12 ± 0.01	0.18 ± 0.009
CWBI-B1419	98.6 ± 0.98	97.2 ± 0.93	97.56 ± 0.83	0.11 ± 0.009	0.11 ± 0.007	0.11 ± 0.001
